# Efficient Processing of Spatio-Temporal Data Streams With Spiking Neural Networks

**DOI:** 10.3389/fnins.2020.00439

**Published:** 2020-05-05

**Authors:** Alexander Kugele, Thomas Pfeil, Michael Pfeiffer, Elisabetta Chicca

**Affiliations:** ^1^Faculty of Technology and Center of Cognitive Interaction Technology (CITEC), Bielefeld University, Bielefeld, Germany; ^2^Bosch Center for Artificial Intelligence, Renningen, Germany

**Keywords:** spiking neural networks, sequence processing, efficient inference, neuromorphic computing, event-based vision

## Abstract

Spiking neural networks (SNNs) are potentially highly efficient models for inference on fully parallel neuromorphic hardware, but existing training methods that convert conventional artificial neural networks (ANNs) into SNNs are unable to exploit these advantages. Although ANN-to-SNN conversion has achieved state-of-the-art accuracy for static image classification tasks, the following subtle but important difference in the way SNNs and ANNs integrate information over time makes the direct application of conversion techniques for sequence processing tasks challenging. Whereas all connections in SNNs have a certain propagation delay larger than zero, ANNs assign different roles to feed-forward connections, which immediately update all neurons within the same time step, and recurrent connections, which have to be rolled out in time and are typically assigned a delay of one time step. Here, we present a novel method to obtain highly accurate SNNs for sequence processing by modifying the ANN training before conversion, such that delays induced by ANN rollouts match the propagation delays in the targeted SNN implementation. Our method builds on the recently introduced framework of streaming rollouts, which aims for fully parallel model execution of ANNs and inherently allows for temporal integration by merging paths of different delays between input and output of the network. The resulting networks achieve state-of-the-art accuracy for multiple event-based benchmark datasets, including N-MNIST, CIFAR10-DVS, N-CARS, and DvsGesture, and through the use of spatio-temporal shortcut connections yield low-latency approximate network responses that improve over time as more of the input sequence is processed. In addition, our converted SNNs are consistently more energy-efficient than their corresponding ANNs.

## 1. Introduction

Spiking neural networks (SNNs) were initially developed as biophysically realistic models of information processing in nervous systems (Rieke et al., 1999; Gerstner et al., 2014), but they are also ideally suited to process data from event-based sensors (Posch et al., 2010; Liu and Delbruck, 2010; Furber et al., 2013; O'Connor et al., 2013; Osswald et al., 2017), and are natively implemented on various neuromorphic computing platforms (Schemmel et al., 2010; Furber et al., 2013; Merolla et al., 2014; Qiao et al., 2015; Martí et al., 2015; Davies et al., 2018). Their sparse and event-driven mode of computation makes them more energy-efficient and faster compared to conventional artificial neural networks (ANNs), and additionally allows for the use of spatio-temporal spike codes to represent complex relationships between features in the network. These hypothetical advantages can, however, only be completely exploited on hardware that supports *fully parallel model execution*, which means that spiking neurons operate independently from each other and their update is solely based on incoming spikes. This is different from typical ANN execution schemes, which update all neurons in a fixed order determined by the network architecture and at fixed discrete time steps.

The goal of this article is to develop a framework for obtaining SNNs that run fully in parallel and achieve high accuracy, low latency, and high energy-efficiency on sequence processing tasks, in particular classifying streams of events from neuromorphic sensors. Sequence processing seems to be a natural fit for the execution mode of SNNs where every neuron has its own dynamics, but in practice it has proven to be very challenging to exploit this property to train SNNs on temporally varying input data. Even more, current state-of-the-art methods for SNN training are unable to yield competitive accuracies compared to ANNs even in the simpler case of static inputs (Pfeiffer and Pfeil, 2018), albeit the gap has become narrower over the past years due to better training algorithms, such as e.g., variants of backpropagation for SNNs (Lee et al., 2016; Wu et al., 2018; Shrestha and Orchard, 2018; Neftci et al., 2019). However, Deng et al. (2020) argue that SNNs in general are put at a disadvantage in tasks designed for ANNs, such as image classification, because of the information loss incurred during conversion of images to spike trains of finite time window length. SNNs should not be expected to outperform ANNs in terms of accuracy on frame-based tasks, but they may be advantageous in terms of memory and compute costs. SNNs should ideally always be evaluated on event-based datasets, where they are able to outperform ANNs by exploiting the spatio-temporal information encoding of event-streams. Consequently, in this article we use only event-based datasets to evaluate our SNN performance and report memory and compute requirements for our networks, as suggested in Deng et al. (2020).

The currently most successful method for obtaining accurate SNNs is to train an ANN with conventional deep learning methods, and convert the resulting ANN architecture and weights into an equivalent SNN, translating analog neuron activations into proportional firing rates of spiking neurons (Cao et al., 2015; Rueckauer et al., 2017). Conversion methods have achieved the best known SNN accuracies for image classification tasks, such as MNIST, but they rely on the assumption that input patterns do not change for some time. This is required because firing rates in each layer need time to converge to their targets derived from ANN activations. Spikes are allowed to propagate instantaneously between layers of the network, since this speeds up convergence of firing rates in deeper layers, and there is no additional temporal information beyond rates encoded in spike trains.

These assumptions are no longer valid when sequence processing tasks are considered, which require networks capable of temporal integration. Temporal integration means that information from different times of the input has to be integrated at a single point in time at the output of the network. In a multi-layer network this means that the network architecture as well as the propagation delays between layers become crucial to control not just what features of the input are computed, but also when information computed in other layers can be used to update the feature computation. Temporal integration is achieved with recurrent or *temporal skip connections*, which not only skip layers in depth-direction of the network, but also bridge time like recurrent connections. Since temporal skip connections, in contrast to recurrent connections, serve as shortcuts in time, and hence, reduce the latency of early approximate network responses, we omit recurrent connections in the following.

Our goal is to obtain SNNs for model-parallel execution on actual neuromorphic systems, which requires assigning non-zero delays to all connections in the network. However, current ANN-to-SNN conversion methods are unable to deal with the case of time-varying inputs or with temporal skip connections with different propagation delays. The main contribution of this paper is to close these gaps by unifying ANN-to-SNN conversion with the recently introduced concept of streaming rollouts (Fischer et al., 2018), thereby greatly extending the applicability of SNN training methods to novel and important classes of applications. Since the inference graph of an SNN determines the way temporal information is being processed, its temporal structure needs already to be taken into account during ANN training (see Section 2.2 for details). In other words, it has to be ensured that information from all required parts of the input sequence and the resulting activations of intermediate layers arrives at the right time at the output neurons both during ANN training and after conversion to SNNs. With this novel method for rolling out and training ANNs before conversion to SNNs we obtain SNNs that efficiently and accurately solve sequence processing tasks, and yield approximate responses as early as possible.

In the following, we describe our methods in detail and show experimental results that emphasize the advantages of our approach for event-based sequence processing tasks.

## 2. Methods

In this section, we describe the task of classifying event-based data streams with spiking neural networks (Section 2.1), and present a recipe for obtaining SNNs to process input sequences on neuromorphic hardware. First, we define the targeted inference graph of SNNs (Section 2.2) and, then, describe how to train (Section 2.3) and convert (Section 2.4) corresponding artificial neural networks (ANNs). Last, we describe how we estimate the energy-efficiency of both approaches in Section 2.5.

### 2.1. Classification With Spiking Neural Networks

We study the task of training an SNN that processes a given input spike sequence **S**_in_ into a discrete target output *y* ∈ {1, …, *C*}, where *C* is the number of available classes. The input **S**_in_ is a multi-dimensional spike sequence of dimensionality *M*, where $S_{\text{in}}^{\left(i\right)}=\left(t_{\text{in},1}^{\left(i\right)},\ldots ,t_{\text{in},n\left(i\right)}^{\left(i\right)}\right)$ defines the spike times of neuron *i* ∈ {1, …, *M*}, and *n*(*i*) ≥ 0 is the number of spikes generated by input neuron *i* in the input sequence. We define $T_{\text{max}}\left({\text{S}}_{\text{in}}\right)=\underset{i=1,\ldots ,M}{\max}t_{\text{in},n\left(i\right)}^{\left(i\right)}$ as the length of the complete sequence, i.e., the time of the final input spike to any input neuron, and we denote by **S**[*t*_0_, *t*_1_] the partial spike train that includes all spikes of **S** between *t*_0_ and *t*_1_. We introduce the shortcut **S**[*t*] = **S**[0, *t*] for all spikes up to time *t*. The output vector *y*(*t*) is computed from the spike trains **S**_out_[*t*] of a defined output layer of the network after seeing all spikes up to time *t*, and can be computed in various ways, e.g., by applying the soft max function to the spike counts of all output neurons.

In our experiments the spiking neurons are simple non-leaky Integrate & Fire (IF) neurons without refractory period, as described in Rueckauer et al. (2017). Every neuron *i* is characterized by its membrane potential *V*_*i*_(*t*), which is updated whenever the neuron receives an input spike from another neuron *j*. In this case we update *V*_*i*_(*t*) ← *V*_*i*_(*t*) + *w*_*ij*_. If *V*_*i*_(*t*) exceeds a threshold voltage *V*_th_ then the neuron sends out a spike and resets its membrane potential by the following subtraction: *V*_*i*_(*t*) ← *V*_*i*_(*t*) − *V*_th_. Rueckauer et al. (2017) analytically show how IF neurons can approximate ANN activations with spike rates. It is possible to use alternative neuron models, e.g., leaky integrate-and-fire, but to date no practical benefits have been demonstrated that would warrant their additional analytical and computational complexity. Hence, we consider only IF models in this paper.

### 2.2. Sequence Processing With Streaming Rollouts

The architecture of the neural network is described by a directed *network graph*, in which nodes correspond to layers of a neural network, and edges represent dependencies between the layers. The goal is to train a network for *sequence processing*, which means the output *t* at any time depends on the entire input sequence **S**_in_[*t*] or at least a *spatio-temporal receptive field* **S**_in_[*t* − τ, *t*] of duration τ. The network needs to be capable of *temporal integration*, i.e., information about the input in the relevant spatio-temporal receptive field must remain present in some nodes, and must be continuously combined with new incoming information. Temporal integration requires a network graph that includes either recurrent or temporal skip connections, as discussed next.

In the setting of an ANN processing an input sequence, a network graph can be rolled out in time in multiple ways (Fischer et al., 2018). The usual convention of *sequential rollouts* is to assume a delay of one time step for recurrent edges, whereas all other edges in the feed-forward direction from input to output are assumed to transport information instantaneously without delay. A mechanism similar to sequential rollouts, although on the granularity of SNN simulation time steps was proposed by Wu et al. (2018) to train SNNs with backpropagation, which allows treating the spatial and temporal domain separately for backpropagation. However, this notion of sequential rollouts is in contrast to the fully parallel execution mode of SNNs, in which all neurons can update their states simultaneously, but information cannot be instantaneously propagated between neurons. Converting an ANN trained with sequential rollouts into an SNN can therefore lead to a mismatch in the way information is being processed over time.

Fischer et al. (2018) proposed an alternative rollout mechanism called *streaming rollout*, in which all edges transport information to the next *rollout frame*. We define a rollout frame as the state of all neurons at a given time point after applying all instantaneous updates within the same frame, as well as updates from delayed connections from the previous time step(s). The streaming rollout is equivalent to introducing an axonal delay *d*_ANN_ of at least one rollout frame to all connections. Each neuron's next state can then be computed exclusively from values computed in the previous rollout frame, which allows fully parallel updates within one rollout frame. In previous conversion approaches, the time to reach good approximations scales with the network depth, because the spiking activity in any layer first needs to converge to a good-enough approximation of ANN activations before the next layer is able to generate precise approximations. This is resolved by the streaming rollout, as all layers approximate the activations in parallel, thereby decoupling the depth from the integration time. We limit our analysis in this paper to a delay of one rollout frame.

Skip connections under streaming rollout translate to *temporal skip connections*, which do not only skip layers in the depth-direction of the network, but also span time. Furthermore, temporal skip connections give rise to early approximate results, and the earliest response is determined by the shortest path between input and output in the network graph. Initial predictions are less accurate, because only a shallower network is used for classification, but getting an early guess is desirable for many tasks that require real-time decisions. Note that the scenario we investigate here is different from the typical sequence processing framework, e.g., NLP or speech, where reasonable accuracy can only be obtained after seeing most of the input. The accuracy improves over time as more frames of the input sequence are processed, and as deeper layers of the network begin to contribute to the prediction.

**Figure 1:**
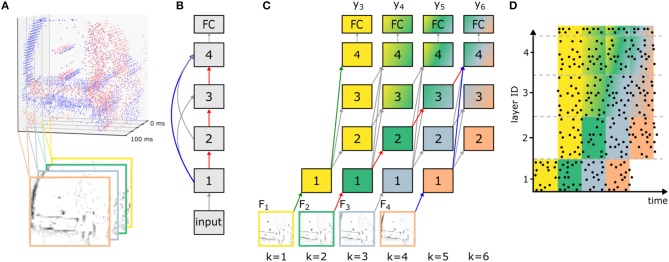
Network rollout and training of ANNs before conversion to SNNs for sequence processing. **(A)** Input frames *F*_*in*_ are generated from event data by averaging ON (red) and OFF (blue) events over time intervals of fixed duration *T*_F_ (here *T*_F_ = 25 ms; in the bottom only OFF events are shown). This example is taken from the N-CARS dataset. **(B)** Network graph of an exemplary feed-forward SNN consisting of an initial convolutional layer (with index 1), one block of three convolutional layers (2 to 4) and a fully-connected (FC) layer that are connected to each other. **(C)** The streaming rollout of the ANN that corresponds to the connectivity chosen in (B) over *K* = 6 rollout frames *k* and *d*_ANN_ = 1 for all connections. Note that the model parallelism required by SNNs is achieved by choosing all connections to span time, i.e., to bridge rollout frames. For networks with skip connections, this results in paths of different length from input *F*_*k*_ to output $y_{k^{\prime }}$ (e.g., red and blue path from *F*_2_ and *F*_4_ to *y*_6_) allowing for spatio-temporal integration of information over the input sequence *F*_*in*_. Nodes in the rollout are numbered like in **(B)** and identically numbered nodes indicate that they share their weights. **(D)** Exemplary activity of an SNN (raster plot) after conversion from a trained ANN as shown in **(C)**. Each row corresponds to a neuron in the SNN and each data point is a single spike. Note that the absence of activity in layers 2–4 in the first rollout frame is caused by axonal delays.

Figure 1 illustrates how streaming rollouts achieve temporal integration for an exemplary network graph (Figure 1B) with *N*_l_ = 4 convolutions and a fully-connected layer (number of blocks *N*_b_ = 1). The temporal shortcuts with *d*_ANN_ = 1 allow for temporal integration over multiple frames in the input sequence. This is illustrated in Figure 1C by assigning a color to each of the four processed input frames *F*_1_, …, *F*_4_, and the mixing of colors indicates which frames provide information to which layer at every time step. For example, the skip connection from layer 1 to 4 causes early activity in the output layer already at *k* = 3, although with reduced accuracy. Multiple paths of different lengths connect the input to the output, with a shortest path (shown in blue) of length 2, and the longest path (in red) of length 4. The difference between the length of the longest and the shortest path determines the size of the spatio-temporal receptive field. In our example, the size of the receptive field is τ = 3 input frames and, hence, the output in Figure 1C is shown as a mix of up to three colors. In Figure 1C at *k* = 6, input data from *k* = 2 and 4 arrive at the same time at the output layer (via the red and blue path, respectively) and can, hence, be jointly used for prediction. In addition, the shortest path between inputs and outputs of such a network (green path) defines the latency, at which a first approximate prediction can be made. Long paths (red path), i.e., deeper networks, allow for better accuracy at the cost of higher computational effort. In addition, the overall energy-efficiency is further increased by regularizing all activations with the L2-norm in order to achieve smaller activations and therefore reduce the number of operations necessary to reach an accuracy level close to the maximum possible. Note that in streaming rollouts newly acquired input frames are immediately processed and fused with pre-processed information from previous inputs to refine the output of the network. Since the computation of all layers in a rollout frame only depends on the outputs of the layers in the last rollout frame, outputs can be computed frame by frame. This is in contrast to other methods for sequence processing, for which multiple input frames are required at once to compute the output of the network (e.g., van den Oord et al., 2016a).

### 2.3. Training of Artificial Neural Networks

In all our experiments, the network graphs follow the DenseNet architecture (Huang et al., 2017) due to two main reasons. First, DenseNets are established network models and achieve competitive results across various applications (Zhu and Newsam, 2017; Huang et al., 2018; Zhang et al., 2018). Second, the dense connectivity between layers, as described in the following, results in streaming rollouts, in which the output is updated every time step. In each block of a DenseNet, every layer is connected to all previous layers. The blocks are connected by transition layers that reduce the resolution via pooling. The last layer is composed of global average pooling and a fully-connected layer for classification. Throughout this study, we use network graphs with *N*_b_ = 3 blocks, all other hyperparameters and a full schematic of a two-block DenseNet can be found in Appendix A.

For the streaming rollout of the above network graph, the temporal window τ is limited by the depth of the network *D* = *N*_l_*N*_b_+1. This explicit restriction to a finite temporal window allows choosing a network architecture that matches the temporal scale of the specific problem at hand. Furthermore, the latency from first input to output in streamingly rolled out DenseNets is *N*_b_ rollout frames, which is typically shorter than the latency of *D* for recurrent networks that utilize all *D* layers for each prediction. These temporal skip connections in streamingly rolled out DenseNets allow for fast approximate predictions that are refined over time. For our datasets, we saw an increase in our accuracy when replacing regular dropout with spatial dropout (Tompson et al., 2015) and using convolutions with weight kernels of spatial size 3 × 3 instead of 1 × 1 for the transition layers (for further hyperparameters, see Appendix A).

For the training of ANNs with streaming rollouts, event-based input sequences first need to be converted into sequences *F*_*in*_ of *N* so-called *input frames* *F*_*k*_ (e.g., see Figures 1A,C). The input spike sequences **S**_in_ are divided into *N* equally sized time intervals of length *T*_F_ = *T*(**S**_in_)/*N*, and for each interval we compute the sum of all spikes, which is used as the input to the ANN. Since event-based vision sensors distinguish between ON and OFF events, we compute two separate channels per input frame.

For a given sequence of input frames *F*_*in*_ we use streaming rollouts to compute the activations of all ANN units over time, and apply backpropagation-through-time (Werbos, 1990) to train the weights of the network. To consider all *N* input frames *F*_*k*_ in *F*_*in*_ with *k* ∈ {1, …, *N*} with the shortest possible rollout, the last network output *y*_*k*_ of the rollout is connected to the last input frame *F*_*N*_, via the shortest path *l*_s_. This results in rollouts with *K* = *N* + *l*_s_ rollout frames and as many outputs *y*_*k*_ (with *k* ∈ {*l*_s_, …, *K*}) as inputs (for an example, see Figure 1C). For every dataset, the number of rollout frames *K* is determined by τ, the length of the temporal window. If the number of input frames *N* is smaller than τ, these input frames are evenly distributed over the available τ input slots of the fixed rollouts.

The optimization objective is to minimize the categorical cross entropy *L* over all predictions *y*_*k*_ of the network outputs,

(1) $$\begin{array}{r} L=\overset{K}{\underset{k=l_{\text{s}}}{\sum }}-a_{k}{ŷ}_{k}\log\left(y_{k}\right) \end{array}$$

where ŷ_*k*_ are the one-hot class labels and *a*_*k*_ are factors to trade off between early and late accuracy. These factors will be discussed in detail in Section 3.2. As we are considering classification problems, the target class label is the same for each output, i.e., ŷ_*k*_ = ŷ ∀ *k*. Observe that ŷ_*k*_ log(*y*_*k*_) is a scalar product and since ŷ_*k*_ is a one-hot vector, only one term is non-zero. In all layers, we apply weight decay as regularization, and activation decay for increased sparsity. For parameterizations and further details, see Appendix A.

### 2.4. ANN-to-SNN Conversion

After training the streaming rollout of the ANN, the architecture and weights of the ANN are translated into an equivalent SNN for energy-efficient inference. We closely follow the conversion method described by Rueckauer et al. (2017), who proved that, under the assumption of ReLU activations and IF neurons, the firing rate *r*_*i*_ of a spiking neuron *i* becomes proportional to the activation *a*_*i*_ of the corresponding neuron *i* in the ANN. Hence, for the same input, the output firing rates of the SNN approximate the ANN output activations, and the approximation error decreases with simulation time of the SNN. In order to speed up this approximation, the authors proposed a weight normalization scheme to fully use the dynamic range of the spiking neurons determined by their maximum firing rate.

In this article we go beyond the mechanisms described Rueckauer et al. (2017), and apply ANN-to-SNN conversion to a network rolled out in time using streaming rollouts (see section 2.2 and Figure 1C), thereby allowing to address sequence processing tasks. Two levels of temporal integration have to be considered for the SNN: First, for every rollout frame, ANN activations are approximated by firing rates, which happens in the time interval defined by *T*_F_. Therefore, we have to set the duration of a rollout frame long enough for firing rates to converge to their target rates. Second, skip connections in streaming rollouts allow temporal integration of information. For example, the red and blue path in Figure 1C are arriving at the same time at layer 4 at *k*, because each connection has delay *d*_ANN_ = 1. Consequently, axonal delays of connections in SNNs have to be set such that information propagates through the network as predefined by the rollout of the ANN. If ANN activations in each rollout frame are approximated by *n*_sf_ simulation steps in SNNs, the delay in SNNs has to satisfy *d* = *n*_sf_ · *d*_ANN_ = *n*_sf_. Additionally, to prevent neurons from being inactive for too long after receiving a sustained negative input during one rollout frame we use a lower bound on the membrane potential. It is expected that the output rate of a neuron changes smoothly with its input rate. Assuming that the input changes slowly over time, the membrane voltage stored at the end of one rollout frame will be a good initialization for the next rollout frame. In addition, the time until the rate approximation is sufficiently good decreases for each additional rollout frame. The limitation is the resolution with which the rates have to be approximated. A particular advantage of using skip connections is that the time required for information to propagate from input to output is determined by the shortest path *l*_s_. This rate of change from input to output is usually higher than for networks using recurrent instead of skip connections, since for these the shortest path equals to the full depth of the network (*l*_s_ = *D*), i.e., information needs to propagate through all layers.

Classification outputs in the final layer of the spiking network are computed as $y\left(t\right)=\text{arg max}\left(\overset{t}{\underset{t^{\prime }=t-T_{\text{F}}}{\sum }}{\text{S}}_{\text{out}}\left[t^{\prime }\right]\right)$ by summing all weighted input spikes to each neuron over the time interval *T*_F_ and taking the arg max of this vector. This allows faster adaptation of predictions, does not need an external stimulus, and handles the case when both output neuron activations are negative.

One important aspect of a classification method is the latency between inputs and outputs, especially in scenarios with critical real-time requirements. A direct approach would be to measure the wall-clock time required to execute the SNNs. However, the execution time of SNNs strongly depends on the used hardware system. In order to disentangle this dependency we introduce the hardware-agnostic measure of simulation steps per frame *n*_sf_. In a time-stepped SNN simulator, each frame of a sample is used as input for *n*_sf_ steps. Then, the actual wall-clock time depends on, first, the throughput of the SNN simulator/emulator *f* in simulation steps per second and, second, the time needed to accumulate one frame *T*_F_ (see Figure 1). If a new frame is accumulated while the network is executed and *fn*_sf_ ≤ *T*_F_ holds, the system runs in real time.

The core idea of ANN-to-SNN conversion is to achieve a linear mapping between activations of the ANN neurons and spike rates of the SNN neurons. Neurons should not saturate, i.e., the rate after mapping should not exceed one spike per simulation step. Therefore, the activations have to be rescaled, which can be achieved by rescaling weights and biases in each layer by a scalar factor. The employed robust scheme scales the parameters of each layer by a predefined percentile of the training set activations, as described in Rueckauer et al. (2017). Rescaling by a percentile of the activations instead of the maximum activation leads to some neurons saturating (they should spike more than once per simulation step), but increases the overall activity in the network, leading to faster propagation of information and therefore reduces the latency between input and output. Additionally, the authors of Rueckauer et al. (2017) see an increase in accuracy when choosing a percentile as scaling factor instead of the maximum activation. It should be noted that this method is not dependent on the layers used, considers also concatenations of layers and only needs one forward pass to rescale all layers. For our approach, we have to consider that activations change over time. We calculate the percentile over all activations over time, but still for each layer separately. In contrast to the original work, we also rescale the weights of Average Pooling layers by a percentile of the activations and observe an increase in top accuracy.

### 2.5. Energy-Efficiency and Number of Operations

In order to compare the energy-efficiency of ANNs and SNNs we use the same metric as in Rueckauer et al. (2017), i.e., we measure the average number of operations over all samples in the used dataset split during inference. The number of operations is calculated differently for ANNs and SNNs, due to the difference in their neuron models. As discussed by e.g., Thakur et al. (2018) and Pfeiffer and Pfeil (2018), many different neuron models exist for SNNs depending on the desired biological plausibility and complexity. In this study, we use the IF neuron model as described in Section 2.1 to match the method for ANN-to-SNN conversion introduced by Rueckauer et al. (2017). Comparing a forward pass from layer *l* to layer *l* + 1 with activations *a*^*l*^ and connection weights $W_{ij}^{l}$ in ANNs

(2) $$\begin{array}{r} a_{i}^{l+1}=\text{ReLU}\left(\underset{j}{\sum }W_{ij}^{l}a_{j}^{l}\right). \end{array}$$

to the rate approximations of these activations *a*_*l*_ in SNNs as described in Section 2.1, we follow Rueckauer et al. (2017) and define the number of operations for ANNs and SNNs as follows.

For ANNs, operations are defined as the sum of all multiply-add computations, and for SNNs, operations are defined as synaptic operations, i.e., the sum of all spikes processed by all neurons. The number of ANN operations is constant across samples and rollout frames and only depends on the size of the input frame. In contrast, for SNNs, the number of operations depends on how many spikes are generated during the execution of the network. The overall number of spikes typically grows with the number of simulation steps *n*_sf_ and the magnitude of ANN activations, while it decreases with the sparsity of activations. Thus, a smaller number of simulation steps *n*_sf_ in SNNs leads to better energy-efficiency, but also to a less accurate approximation of ANN activations, potentially reducing accuracy. Generally, real-valued multiply-add operations in ANNs are computationally more expensive than synaptic operations in SNNs, but on the other hand memory accesses are more structured for ANNs. This trade-off varies between different accelerators and neuromorphic chips. As an estimate, the energy per multiply-add operation for a recent FPGA architecture (Manolopoulos et al., 2016) is about 555–1295.4 pJ, while for neuromorphic devices, a synaptic operation consumes only 2.8–360 pJ (Thakur et al., 2018). Observe, that our definition of simulation steps per frame *n*_sf_ is related to the number of simulation steps *T*_tot_ in Rueckauer et al. (2017) and Deng et al. (2020) by the number of rollout frames *K* as *T*_tot_ = *n*_sf_·*K*, i.e., either one can be used to quantify the tradeoff between energy-efficiency and accuracy.

## 3. Results

In this section we demonstrate fast, accurate, and energy-efficient inference with SNNs on five different sequence processing tasks. First, a toy dataset is used to illustrate the concept of temporal integration via streaming rollouts in ANNs, and shows the energy-efficiency of converted SNNs (Section 3.1). Second, we apply our approach to event data recorded by an event-based camera in real-world driving scenes (Section 3.2) and showcase the trade-off between the latency of network responses, the classification accuracy, and energy-efficiency. Finally, we demonstrate state-of-the-art performance on the established N-MNIST, CIFAR10-DVS, and DvsGesture benchmarks for classification on event-streams (Sections 3.3 to 3.5).

### 3.1. Moving Rectangles

**Figure 2:**
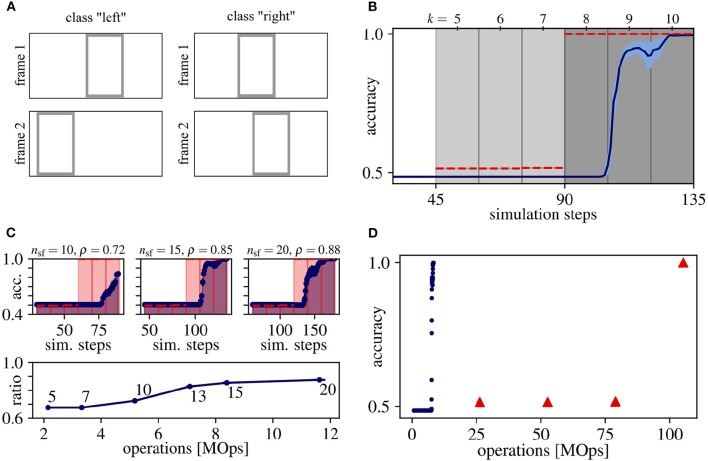
Results for the moving rectangles dataset. **(A)** Samples for each class. **(B)** Average classification accuracy over rollout frames and corresponding simulation steps for ANN (red dashed line) and SNN (blue solid line) with the standard error of the mean in lightly colored areas. Vertical gray lines separate rollout frames and the shading of the background indicates, which image of the input sequence *F*_*in*_ is seen by the network output *y*: Light gray for the first and dark gray for both input frames. Note that the first prediction occurs at the fourth rollout frame after input onset (simulation step = 45), since the shortest path from input to output has length *l*_s_ = 4. **(C)** Determining the trade-off between accuracy and energy-efficiency. (Top) Same as **(B)**, but measured on 1,280 random samples of the training set and for three different values of *n*_sf_. (Bottom) The ratio ρ between the area under curve of the ANN (shaded red) and SNN (shaded blue) for different values of *n*_sf_ over the number of operations. The number next to each datum is its respective number of simulation steps per frame *n*_sf_. The accuracy ratio ρ saturates at *n*_sf_ = 15, which we therefore consider as a good trade-off between accuracy and energy-efficiency. **(D)** Average classification accuracy over the number of operations for ANN (red) and SNN (blue). For all data points in **(B–D)**, averages over 10 trials are plotted. Standard error of the means are always plotted, but sometimes too small to be visible.

This synthetic dataset consists of sequences composed of two frames containing one rectangle each, and the task is to determine whether the rectangle has moved to the left or right in the second image. See Figure 2A for an example of both classes and Appendix A.1 for more details. This frame-based toy dataset is a minimal example of temporal integration, because the direction of movement can only be inferred from the complete sequence, but not from a single image. For this example, we use the network graph as described in Section 2.3 with *N*_l_ = 1 that results in streaming rollouts with a spatio-temporal receptive field of duration τ = 3. To demonstrate the effect of temporal integration, we train this rollout with τ = 6 input frames (for details, see Section 2.3), of which the first and second half (three input frames each) comprises the first and second image of the pair of moving rectangles, respectively.

During ANN inference, the predictions of the first three outputs of this rollout are on chance level (see data points in the area with light gray shading in Figure 2B), because only the first τ = 3 input frames comprising the first rectangle are seen by these outputs. The network outputs at rollout frames *k* = 8 to 10 (dark gray shading) retrieve information from both frames of the input pair and, hence, can integrate this information to perfectly classify the movement direction of the rectangle. Note that for the chosen network graph and streaming rollout, the first response of the network occurs at *k* = 5, which reflects the length and the temporal delay *l*_s_ = 4 of the shortest path between input and output of the rollout.

Figure 2B shows that the SNN's accuracy follows that of the ANN and results in comparable overall performance. However, the SNN accuracy is lagging behind the ANN accuracy in Figures 2B,C, by at least one rollout frame. Multiple reasons could cause this lag: First, the SNN accuracy is calculated by averaging predictions over the last *n*_sf_ simulation steps. Second, accuracies could be further delayed by information that is stored in the values of the membrane potentials and is carried from one to the next rollout frame, which can slow down the convergence of the approximation of firing rates.

To be able to execute the SNN, we have to determine the number of simulation steps per frame *n*_sf_. Observe, that this hyperparameter comes with a trade-off between accuracy and energy-efficiency: With increasing *n*_sf_, the rate approximation error decreases, leading to higher accuracies but also to a higher number of operations, decreasing energy-efficiency. In order to determine a good trade-off between accuracy and energy-efficiency, we sweep over different values for *n*_sf_ using 1,280 randomly chosen samples from the training set. The area under the curve is calculated for both the ANN and SNN accuracy over simulation steps, and the *accuracy ratio* ρ is calculated as the ratio between these areas. The difference between the accuracies of ANNs and SNNs decreases, i.e., ρ increases, with larger *n*_sf_ (see Figure 2C). Note that reaching ρ = 1 implies that ANN activations would have to be approximated by SNN firing rates instantaneously, i.e., within one simulation step, which is very unlikely in practice. The accuracy ratio ρ starts to saturate at *n*_sf_ = 15 simulation steps per rollout frame and, consequently, we consider this value as a good trade-off for our experiments in Figure 2D.

In terms of efficiency, achieving the peak accuracy of 100% for this task requires 8.4 ± 0.6 MOps operations in the SNNs, which is approximately a factor of 13 lower compared to their ANN counterparts (105 MOps, see Figure 2D).

### 3.2. N-CARS

In this section, we apply our methods to real-world event-based vision data from driving scenes, for which the task is to classify the presence of a car in the recorded scene (Sironi et al., 2018). N-CARS uses event streams with continuous spike times for ON and OFF events that are triggered by positive and negative changes in light intensity, respectively. For this experiment, we choose ANN rollouts with *N*_l_ = 5 layers per block resulting in τ = 16 input slots for the rollout and a spatio-temporal receptive field of duration τ = 16 (for details, see Appendix A).

**Figure 3:**
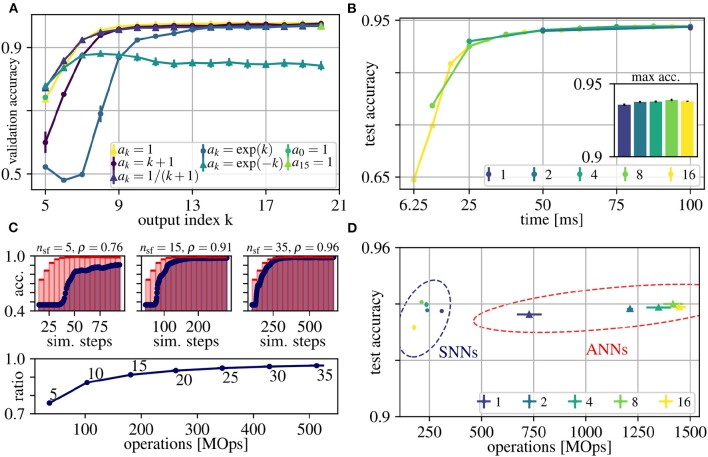
Results for the N-CARS dataset. **(A)** Average validation accuracies over the index *k* of the outputs of the network rollouts. Different choices for weighting these outputs in the loss function are depicted with different colors (for details, see legend and Section 3). **(B)** Average accuracies of ANNs over real time of the input sequences for different values for *N* (see legend). The inset shows the maximum accuracies. **(C)** Determining the trade-off between accuracy and energy-efficiency. (Top) Accuracy of ANN and converted SNN over simulation time for *N* = 16 and *n*_sf_ ∈ {5, 15, 35}. Note that the first prediction occurs at the fourth rollout frame after input onset, since the shortest path from input to output has the length *l*_s_ = 4. (Bottom) The ratio ρ between the area under curve of the ANN (shaded red) and SNN (shaded blue) for different values of *n*_sf_ over the number of operations. The number next to each datum is its respective number of simulation steps per frame *n*_sf_. The accuracy ratio ρ saturates at *n*_sf_ = 15, which we therefore consider as a good trade-off between accuracy and energy-efficiency. **(D)** Average peak accuracies over number of operations for the ANNs of **(B)** and the converted SNNs [same color coding as in **(B)**]. For all shown data, the error of the mean values are plotted after averaging over 10 trials, but are often too small to be seen.

Our goal is to obtain good early predictions without sacrificing accuracies at later outputs (as already discussed in Section 2.2). This enables us to use early outputs for fast but relatively inaccurate results and later outputs for slower results with higher accuracy. This trade-off between early and late performance can be tuned by the factors *a*_*k*_ of the loss function, which weight the losses from outputs at rollout frames *k* = 5 to *k* = 21 (Eq. (1)). The index starts at *k* = 5, because the shortest path through the network is *l*_s_ = 5. In order to determine a good choice for *a*_*k*_ that achieves good early and late performance, we evaluated seven different options. Figure 3A shows resulting accuracies for each output *k* separately, after training, for the following proposals of sets of *a*_*k*_:

- *a*_*k*_ = 1: Uniform weighting: the factor is identical for all *k*, such that early and late accuracy is considered equally important
- *a*_*k*_ = *k* + 1: Linearly increasing weighting: emphasizing late performance
- *a*_*k*_ = exp(*k*): Exponentially increasing weighting: even stronger emphasis on late performance
- *a*_0_ = 1, others 0: Only consider first output
- *a*_*k*_ = 1/(*k* + 1): Moderately decreasing weighting: Emphasizing early performance
- *a*_*k*_ = exp(−*k*): Exponentially decreasing weighting: even stronger emphasis on early performance
- *a*_15_ = 1, others 0: Only consider last output

In practice, we normalize each set of *a*_*k*_ such that $\underset{k}{\sum }a_{k}=1$, to avoid influencing the learning rate. Note that we share weights over time and, consequently, the very same weights have to fulfill multiple objectives at once, which could potentially deteriorate the accuracy of the network outputs. Weighting early outputs higher improves the early accuracy by up to 4.3% compared to constant *a*_*k*_ (77.9 ± 0.2% for exp(−*k*) vs. 73.6 ± 0.8% for *a*_*k*_ = 1). However, increasing the accuracy for early outputs degrades late performance by up to 13.5 % (exp(−*k*)). This effect can be explained by the trade-off between using the available capacity of the network to decrease the loss at early outputs and to provide meaningful features for further processing required to decrease the loss at later outputs. Weighting early outputs much higher than late outputs (exp(−*k*)) shifts this trade-off toward using most of the network capacity for instant classification and suppressing feature generation for later outputs and temporal integration. In all cases in which the late performance was prioritized (including *a*_*k*_ = 1), the maximum accuracies are similar to each other (< 0.04% difference). In conclusion, a uniform weighting *a*_*k*_ = 1 represents a good trade-off between early and late performance indicating synergies between generating rich features required to achieve peak performance and generating sufficient features for immediate classification. Other choices for the weighting *a*_*k*_ either result in lower peak or lower early accuracy (see Figure 3A).

For ANN training, each event-stream of duration 100 ms is divided into *N* input frames and in the following we investigate the impact of different choices for *N* on accuracy, latency and energy-efficiency. The value of each pixel of these input frames is computed by averaging the firing rate during the resulting time intervals *T*_F_ (see also Section 2.3). The sampling frequency 1/*T*_F_ of event streams has to be chosen high enough to resolve the temporal information present in the input sequence and to achieve higher spatial sparsity of the generated input frames, but higher frequencies also cause lower signal-to-noise ratios due to fewer events per input frame. In real-time scenarios, higher sampling frequencies, i.e., smaller *T*_F_, result in more frequent and earlier network responses (see Figure 3B). For example for *N* = 16, the first prediction occurs at *T*_F_ = 100 ms/16 = 6.25 ms, while for *N* = 1 the first prediction occurs at *T*_F_ = 100 ms. Note that for simplicity, we assume the computation of the network output *y* to be instantaneous in Figure 3B, and hence, the shown time axis reflects the time scale of the input sequence. Using the classification accuracies as evaluation criteria, we find *N* = 16 to reach the same accuracy with a higher temporal resolution as other frame intervals (see Figure 3B). For all *N*, we observe that the first network outputs (leftmost data points in Figure 3B) are already above chance level, although they only see the first input frame via the shortest path of the network. These early approximate predictions are then refined in later rollout frames, in which deeper networks can integrate information over multiple input frames. Overall, although sampled with different frequencies, the peak accuracy is almost the same across different *N*. This indicates that, for the N-CARS dataset, the information encoded in time is much less important than the spatial information. We conclude that the information present in the first 25 ms is already sufficient for a successful classification close to peak accuracy. This can be observed in the example given in Figure 1A, where the shape of the car can be distinctively identified after 25 ms.

After conversion, we have to choose the simulation steps per rollout frame *n*_sf_, which significantly influences the accuracy and energy-efficiency of our network. As in Rueckauer et al. (2017), the approximation error of activations in ANNs by firing rates in SNNs increases over time. However, in our case this only holds on a per-frame basis, i.e., in our case the approximation error depends on the number of simulation steps per frame *n*_sf_. Like for the moving rectangles dataset, we measure the accuracy ratio ρ over *n*_sf_ to find a good trade-off between energy-efficiency and accuracy. The accuracy ratio ρ starts to saturate at *n*_sf_ = 15 simulation steps per rollout frame (see Figure 3C) and, consequently, we consider this value as a good trade-off for our experiments in Figure 3D. For smaller *n*_sf_, mostly the early accuracy (e.g., between simulation step 50 and 150 in Figure 3C) suffers, which can be explained as follows: First, SNNs perform worse than ANNs, because for early network outputs, spikes are present only in the short paths from input to output of the networks. Consequently, the overall spiking activity is low, slowing down the convergence of the firing rate approximations. Second, neurons are initialized with lowest (remember that *V* ∈ [0, 1]) membrane voltage *V*(0) = 0, and, hence, it takes a few simulation steps until the neuron can spike. Third, transmitting information from one layer to the next requires at least one simulation step, resulting in a linear increase of the delay from input to output with the number of layers. In our case, the shortest path from input to output has to pass four layers and, hence the information is delayed by four simulation steps, such that the ideal case of *r* = 1 is impossible to reach. Converting ANNs to SNNs and using our choice for *n*_sf_ during the simulations of SNNs, results in accuracies of SNNs that are comparable to their corresponding ANNs, but requiring less energy. Furthermore, the energy-efficiency increases with the number of input frames *N*, up to an 8-fold factor for *N* = 16. Overall, to the best of our knowledge, both our ANNs and SNNs achieve the currently best results on the N-CARS dataset (see Table 1). In addition, our network has 126 378 parameters for *N*_l_ = 5, which is significantly lower than the other approaches.

**Table 1:** Average accuracies for the N-CARS dataset for ANNs and SNNs (10 rials each).

**N-CARS**	**Acc**.	**# params**	**# ops **[MOps]****
HATS/linear SVM (Sironi et al., 2018)	90.2	–	–
Rec. U-Net+CNN (Rebecq et al., 2019)	91.0	>10^6^	–
ResNet-34 (Gehrig et al., 2019)	92.5	10^7^	–
Streaming rollout ANN (**ours**)	94.00(±0.05)	10^5^	1,420(±47)
Converted SNN (**ours**)	**94.07**(±0.05)	10^5^	212.9(±2.5)

*Numbers in parentheses are standard errors of the mean values. # params are the number of parameters, i.e., the number of weights and biases of the network. # ops are the number of operations as defined in section 2.5. A minus sign indicates that the number of parameters or operations could not be estimated from the information available in the respective reference. The highest accuracy is highlighted in bold*.

Since objects, e.g., cars, in the N-CARS dataset only slightly move during the short duration of the recordings, the frames of the input sequence are similar to each other (e.g., see data sample in Figure 1A). For almost static input, intermediate activations do not vary between rollout frames, and, hence, activations of ANNs can be approximated over multiple rollout frames in SNNs. The low number *n*_sf_ = 15 of simulation steps per rollout frame supports this hypothesis. This might also explain, why the SNN peak performance is above the ANN performance (see Table 1), since SNNs intrinsically average activations over rollout frames and may thereby increase the signal-to-noise ratio of the network outputs.

### 3.3. N-MNIST

N-MNIST (Orchard et al., 2015) is a widely used benchmark dataset for SNNs, which allows a comparison of our approach to various alternatives. Each sample in the N-MNIST dataset consists of a digit from the MNIST dataset projected onto a white wall and recorded with an event-based vision sensor, while performing three quick movements (saccades). The challenge is that as the digit moves the active pixels overlap, which means that averaging events over longer time periods results in blurred images, and classification becomes more difficult. We use the same network as for N-CARS, but with *N* = 32 input frames and a growth factor *g* = 15, which was determined by sweeping over *g* ∈ {9, 12, 15, 18}. Competitive results compared to state-of-the art methods are achieved (see Table 2) and the SNN after conversion is ~7 times more energy-efficient than its ANN counterpart.

**Table 2:** Average accuracies for the N-MNIST dataset for ANNs and SNNs (10 trials each).

**N-MNIST**	**Acc**.	**# params**	**# ops **[MOps]****
SNN with backprop (Lee et al., 2016)	98.66	2·10^6^	–
SNN with backprop (Wu et al., 2019)	99.53	2·10^6^	–
HATS/linear SVM (Sironi et al., 2018)	99.1	–	–
Rec. U-Net+CNN (Rebecq et al., 2019)	98.3	>10^6^	–
Streaming rollout ANN (**ours**)	**99.56**(±0.01	3·10^5^	3,500(±360)
Converted SNN (**ours**)	99.54(±0.01)	3·10^5^	460(±38)

*Numbers in parentheses are standard errors of the mean values. Columns like in Table 1. The highest accuracy is highlighted in bold*.

As the number of parameters is not directly listed in the work we compare to, we estimate them from their experiment description: Rebecq et al. (2019) use a U-Net + ResNet18, which typically has 10^6^ to 10^7^ parameters. Gehrig et al. (2019) use a ResNet-34, which has ~10^7^ parameters. In Lee et al. (2016), three layers with (2312, 800, 10) neurons are used, resulting in 1,857,600 parameters. In Wu et al. (2019), they list different network sizes but we expect their best result to be from their largest network listed in Table 1 in their paper. Each convolutional layer has *C*_in_*C*_out_*k*_*x*_*k*_*y*_ parameters, with *C*_in/out_ the number of input/output channels and *k*_*i*_ the kernel sizes. In total, we count 2,840,704 parameters. Our approach has 319,890 parameters for *N*_l_ = 5, which is significantly lower than the other approaches. Sharing weights over time and taking temporal integration into account through our rollout mechanisms allows reaching state-of-the-art accuracy with a small memory footprint.

### 3.4. Cifar10-DVS

The CIFAR10-DVS dataset (Li et al., 2017) consists of 10,000 images extracted from the popular CIFAR-10 dataset. Each of the 10 classes is represented by 1,000 images. Each of these images is scaled up and moves on a diamond-shaped trajectory on a screen. The scene is recorded by a DVS128 sensor for 1.298 ± 0.040 s (mean and standard deviation over all samples) corresponding to 6 repetitions of the trajectory. The monitor's refresh rate of 60 Hz is filtered out of the event stream after recording. The dataset is split randomly into a training (90%) and test (10%) set while maintaining the balance of classes in each set. Then, the training set is further randomly split into a validation (20%) and new training (80%) set. After each training epoch, the accuracy on the validation set is calculated to determine the best model to be used for testing.

The data is pre-processed by cutting out the first 1.3 s of the event stream and splitting each sample into 48 frames resulting in *T*_F_ = 1.3/48 = 27.08 ms. Each edge of the diamond shape is, therefore, resolved by 48/6/4 = 2 frames. To enable faster training and inference, the spatial resolution of each frame is reduced from 128 × 128 pixels to 32 × 32 pixels by bilinear interpolation. We use the same hyperparameters for the network architecture and training as for N-CARS and N-MNIST, but optimize the growth factor by training networks with *g* ∈ {9, 12, 18, 22} and evaluating their accuracy on the validation set. Networks with *g* = 18 result in the best mean accuracy on the validation set, resulting in 480,852 parameters. The ANN-to-SNN conversion is done like for N-CARS and N-MNIST and *n*_sf_ = 60 simulation steps are found to be a good trade-off between accuracy and energy-efficiency. The accuracy of our approach is better than of any other approach for ANNs and SNNs reported to date, and our SNNs require 5-fold less operations than their corresponding ANNs (see Table 3).

**Table 3:** Average accuracies for the CIFAR10-DVS dataset for ANNs and SNNs (10 trials each).

**CIFAR10-DVS**	**Acc**.	**# params**	**# ops **[MOps]****
HATS/linear SVM (Sironi et al., 2018)	52.4	–	–
SNN with backprop (Wu et al., 2019)	60.5	2·10^6^	–
Streaming rollout ANN (**ours**)	**66.75**(±0.22)	5·10^5^	8,800(±1,300)
Converted SNN (**ours**)	65.61(±0.20)	5·10^5^	1,551(±65)

*Numbers in parentheses are standard errors of the mean values. Columns like in Table 1. The highest accuracy is highlighted in bold*.

### 3.5. DvsGesture

DvsGesture (Amir et al., 2017) is an action recognition dataset, where multiple participants performed 11 different gestures under varying lighting conditions. The gestures have an average duration of 6.5 ± 1.7 s and are recorded with a DVS128 sensor. The last class is an arbitrary gesture that each participant came up with. Because this class is not clearly defined, we train networks both with and without this additional target class, which has also been done in the approaches we compare to. We use the original dataset split of (Amir et al., 2017) and generate a validation set by randomly selecting 10% of the training set.

To simplify training and testing we follow the approach by Shrestha and Orchard (2018) and use only the first 1.5 s of each sample, which still contains multiple repetitions of the gesture. We split each sample into 240 frames corresponding to a frame interval of *T*_F_ = 240/1.5 = 6.25 ms. As for CIFAR10-DVS, we reduce the spatial dimension of each frame from 128 × 128 to 32 × 32 pixels. Inspired by Amir et al. (2017), we use stacks of 10 consecutive frames as input for each rollout frame in both ANNs and converted SNNs, such that each of the 24 inputs has 20 channels (as each frame has an on and off channel). This enables temporal integration over longer time scales without introducing motion blur in the individual frames. Note that the frequency of predictions is reduced to only every ten frames, i.e., every 10·6.25 ms = 62.5 ms. We use the same hyperparameters as for CIFAR10-DVS, except for the growth factor. We sweep over *g* ∈ {6, 9, 12, 15, 18} and find *g* = 9 to perform best for 10 classes and *g* = 12 for 11 classes. Our networks therefore have 476,460 and 821,820 parameters, respectively. ANN and SNN accuracies are on par with other state-of-the-art approaches (Table 4). For our SNNs, the number of operations is ~12.5 times lower than for the corresponding ANNs.

**Table 4:** Average accuracies for the DvsGesture dataset for ANNs and SNNs (10 trials each).

**DvsGesture**	**Acc**.	**# params**	**# ops **[MOps]****
**10 CLASSES**			
SNN on TrueNorth (Amir et al., 2017)	96.7	1.5·10^6^	–
SNN with backprop (Shrestha and Orchard, 2018)	93.64(±0.49)	–	–
PointNet-like ANN (Wang et al., 2019)	97.08	–	–
Streaming rollout ANN (**ours**)	**97.16**(±0.11	5·10^5^	8,150(±740)
Converted SNN (**ours**)	96.97(±0.17)	5·10^5^	651(±43)
**11 CLASSES**			
SNN on TrueNorth (Amir et al., 2017)	94.59	1.5·10^6^	–
PointNet-like ANN (Wang et al., 2019)	95.32	–	–
Streaming rollout ANN (**ours**)	**95.68**(±0.32	8·10^5^	15.000(±1, 000)
Converted SNN (**ours**)	95.56(±0.14)	8·10^5^	931(±24)

*Numbers in parentheses are standard errors of the mean values. Columns like in Table 1. The highest accuracy is highlighted in bold*.

We calculate the number of parameters of Amir et al. (2017) from their Table 1 as $\text{params}=\overset{16}{\underset{i=1}{\sum }}\text{feat}\left[\text{i}\right]\cdot \text{kerne}{\text{l}}_{\text{x}}\left[\text{i}\right]\cdot \text{kerne}{\text{l}}_{\text{y}}\left[\text{i}\right]\cdot \text{feat}\left[\text{i}-1\right]/\text{groups}\left[\text{i}\right]=1,528,536$. Shrestha and Orchard (2018) do not provide a detailed network description for their DvsGesture experiments.

## 4. Discussion

We have presented a novel way of training efficient SNNs for sequence processing via conversion from ANNs. The crucial observation is the connection between axonal delays in the SNN and the rollout strategy in the ANN. Streaming rollouts of ANNs are shown to be a particularly good fit, as they closely resemble the fully model-parallel execution in SNNs. To unify the two approaches, we introduced several additions to the existing conversion approach, such as a more general weight rescaling scheme, a new way to calculate predictions in the SNN, rescaling of average pooling layers and axonal delays. As a result, we make ANN-to-SNN conversion applicable in a principled manner to input signals changing over time, including general time series and the special case of event-based input data. Due to the fact that the streaming rollout imposes constraints on the ANN during training our approach can be interpreted as a “constrain-then-train” approach for SNNs (Esser et al., 2015; Pfeiffer and Pfeil, 2018), for which the superior training mechanisms available for ANNs are combined with the efficiency of SNN execution.

We identify and highlight in our experiments particular advantages of applying conversion to rolled-out networks. Our proposed training and conversion scheme results in SNNs that efficiently integrate temporal information, provide early approximate network outputs, and achieve state-of-the art results on the N-MNIST, N-CARS, DvsGesture and CIFAR10-DVS datasets with smaller networks than other approaches, and with SNNs that are consistently more energy-efficient than their ANN counterparts. A uniform weighting of the network outputs in the loss function enables good early and late performance compared to other weighting patterns, such that even for the first network output, the prediction is significantly above chance level. Our framework is flexible enough to allow different trade-offs between early and late performance by choosing different weight factors *a*_*k*_. In this study, for the first time, streaming rollouts were applied to realistic and large-scale time series data, and were shown to be competitive with other approaches on multiple widely used event-based vision tasks (see Tables 1–4).

Although we use only delays of one rollout frame in our experiments, in principle, arbitrary delays can be incorporated into the network rollouts. This principle is useful to convert advanced ANN architectures with temporal convolutions (van den Oord et al., 2016b; Bai et al., 2018) that require multiple delays when rolled out. This is a big advantage over previous conversion approaches (e.g., Cao et al., 2015; Rueckauer et al., 2017), which do not take delays of connections into account. For purely feed-forward SNNs on suitable hardware (Farabet et al., 2012; Pérez-Carrasco et al., 2013) a pseudo-simultaneous spread of information, i.e., all delays in the network are zero, is advantageous, but causes de-synchronization if information needs to be integrated over time. Our approach generalizes the work of Diehl et al. (2016), who have shown a conversion approach for Elman-type recurrent networks using fixed delays in the recurrent layer and zero delays for feed-forward connections. Note that although our experiments only show DenseNet architectures and therefore lead to a linear growth of the size of the temporal receptive field with network depth, this is not a general restriction of our approach. More complex network graphs, for example containing temporal convolutions or recurrent connections, lead to a super-linear growth of the temporal receptive field.

Rescaling weights and biases during conversion by using percentiles instead of maximum values as upper limits for ANN activations increases the accuracy. However, the percentile values of activations calculated over all rollout frames may overestimate the size of ANN activations in single rollout frames and would, hence, decrease the effective resolution of the firing rate approximation (for details, see Section 2.4). For example, in our network rollouts, the activity increases with each rollout frame (which does not hold in general). This results in strong overestimations of activations for early network outputs, which in turn increase approximation errors and, hence, decrease accuracy (see e.g., Figure 3C). As the activity increases with more and more paths from input to output of the network contributing to later network outputs, this approximation error decreases until an optimal effective resolution is reached when spiking activity is present in all parts of the network. Adaptively rescaling the SNN weights or firing thresholds could be a solution to alleviate this effect. This can be seen as a kind of homeostasis mechanism that keeps the overall firing rates of SNNs at a constant level.

Instead of simply averaging event rates to obtain input frames, our approach generalizes to using more advanced features for event-based vision, such as time surfaces (Sironi et al., 2018), event spike tensors (Gehrig et al., 2019) or motion-based features (Clady et al., 2017). As use-cases for event-based vision are becoming increasingly challenging (Gallego et al., 2019), and neuromorphic hardware platforms become more mature (DeBole et al., 2019), our approach fills an important gap to provide powerful SNNs ready for deployment on those platforms.

A major goal of our approach is achieving energy-efficiency, which we measure by the number of operations necessary to reach the desired performance. High efficiency during early inference is enabled by temporal skip connections and carefully choosing the weight factors *a*_*k*_ in the loss function to achieve a good early accuracy without deteriorating the later peak accuracy. After ANN-to-SNN conversion, the SNNs are consistently more energy-efficient than their corresponding ANNs, and the achieved relative gain in efficiency is higher than, e.g., reported by Rueckauer et al. (2017). This may be due to the different neural architectures and the increased sparsity of the input in our study. The sparsity of a single frame increases with a decreasing time interval *T*_F_ over which events are accumulated. To further increase the efficiency we ran multiple experiments including quantization and observed interesting dependencies between quantization levels, network architectures, energy-efficiency, and final accuracy. A thorough investigation would exceed the scope of this study and is left for future studies.

In summary, our approach sets a new standard for spiking neural networks for processing spatio-temporal event streams both in terms of accuracy and efficiency. However, in this study, information is encoded with firing rates, the underlying principle of network conversions, and we did not exploit the potential of encoding information with spike times that potentially allow for even more energy-efficient solutions (for an overview, see Pfeiffer and Pfeil, 2018). We are excited to see our results as a competitive baseline for further studies in the direction of spike codes.

## Data Availability Statement

The datasets generated for this study are available on request to the corresponding author.

## Author Contributions

AK and TP designed the study, contributed to the source code, conducted the experiments, and evaluated the results. MP and EC provided the feedback and scientific advice throughout the process. All authors contributed to the final manuscript.

## Conflict of Interest

MP, TP, and AK were employed by Robert Bosch GmbH. The remaining author declares that the research was conducted in the absence of any commercial or financial relationships that could be construed as a potential conflict of interest.

## References

[B1] Amir A. Taba B. Berg D. Melano T. McKinstry J. Di Nolfo C. (2017). A low power, fully event-based gesture recognition system, in Proceedings of the IEEE Conference on Computer Vision and Pattern Recognition (Honolulu, HI), 7243–7252. 10.1109/CVPR.2017.781

[B2] Bai S. Kolter J. Z. Koltun V. (2018). An empirical evaluation of generic convolutional and recurrent networks for sequence modeling. CoRR abs/1803.01271.

[B3] Cao Y. Chen Y. Khosla D. (2015). Spiking deep convolutional neural networks for energy-efficient object recognition. Int. J. Comput. Vis. 113, 54–66. 10.1007/s11263-014-0788-3

[B4] Clady X. Maro J.-M. Barré S. Benosman R. B. (2017). A motion-based feature for event-based pattern recognition. Front. Neurosci. 10:594. 10.3389/fnins.2016.00594 28101001 PMC5209354

[B5] Davies M. Srinivasa N. Lin T. Chinya G. Cao Y. Choday S. H. (2018). Loihi: a neuromorphic manycore processor with on-chip learning. IEEE Micro 38, 82–99. 10.1109/MM.2018.112130359

[B6] DeBole M. V. Taba B. Amir A. Akopyan F. Andreopoulos A. Risk W. P. (2019). Truenorth: accelerating from zero to 64 million neurons in 10 years. Computer 52, 20–29. 10.1109/MC.2019.2903009

[B7] Deng L. Wu Y. Hu X. Liang L. Ding Y. Li G.. (2020). Rethinking the performance comparison between SNNs and ANNs. Neural Netw. 121, 294–307. 10.1016/j.neunet.2019.09.005 31586857

[B8] Diehl P. U. Zarrella G. Cassidy A. Pedroni B. U. Neftci E. (2016). Conversion of artificial recurrent neural networks to spiking neural networks for low-power neuromorphic hardware, in 2016 IEEE International Conference on Rebooting Computing (ICRC) (San Diego, CA), 1–8. 10.1109/ICRC.2016.7738691

[B9] Esser S. K. Appuswamy R. Merolla P. Arthur J. V. Modha D. S. (2015). Backpropagation for energy-efficient neuromorphic computing, in Advances in Neural Information Processing Systems (Montreal, QC), 1117–1125.

[B10] Farabet C. Paz R. Pérez-Carrasco J. Zamarreño C. Linares-Barranco A. LeCun Y.. (2012). Comparison between frame-constrained fix-pixel-value and frame-free spiking-dynamic-pixel convnets for visual processing. Front. Neurosci. 6:32. 10.3389/fnins.2012.00032 22518097 PMC3324817

[B11] Fischer V. Koehler J. Pfeil T. (2018). The streaming rollout of deep networks-towards fully model-parallel execution, in Advances in Neural Information Processing Systems 31, eds Bengio S. Wallach H. Larochelle H. Grauman K. Cesa-Bianchi N. Garnett R. (Montreal, QC: Curran Associates, Inc.), 4039–4050.

[B12] Furber S. B. Lester D. R. Plana L. A. Garside J. D. Painkras E. Temple S. (2013). Overview of the spinnaker system architecture. IEEE Trans. Comput. 62, 2454–2467. 10.1109/TC.2012.142

[B13] Gallego G. Delbrück T. Orchard G. Bartolozzi C. Taba B. Censi A. (2019). Event-based vision: a survey. CoRR abs/1904.08405. 10.1109/TPAMI.2020.3008413 32750812

[B14] Gehrig D. Loquercio A. Derpanis K. G. Scaramuzza D. (2019). End-to-end learning of representations for asynchronous event-based data, in The IEEE International Conference on Computer Vision (ICCV) (Seoul). 10.1109/ICCV.2019.00573

[B15] Gerstner W. Kistler W. M. Naud R. Paninski L. (2014). Neuronal Dynamics: From Single Neurons to Networks and Models of Cognition. Cambridge: Cambridge University Press 10.1017/CBO9781107447615

[B16] Huang G. Liu S. van der Maaten L. Weinberger K. Q. (2018). Condensenet: an efficient densenet using learned group convolutions, in The IEEE Conference on Computer Vision and Pattern Recognition (CVPR) (Salt Lake City, UT). 10.1109/CVPR.2018.00291

[B17] Huang G. Liu Z. Weinberger K. Q. (2017). Densely connected convolutional networks, in The IEEE Conference on Computer Vision and Pattern Recognition (CVPR) (Honolulu, HI). 10.1109/CVPR.2017.243

[B18] Lee J. H. Delbruck T. Pfeiffer M. (2016). Training deep spiking neural networks using backpropagation. Front. Neurosci. 10:508. 10.3389/fnins.2016.00508 27877107 PMC5099523

[B19] Li H. Liu H. Ji X. Li G. Shi L. (2017). Cifar10-dvs: an event-stream dataset for object classification. Front. Neurosci. 11:309. 10.3389/fnins.2017.00309 28611582 PMC5447775

[B20] Liu S.-C. Delbruck T. (2010). Neuromorphic sensory systems. Curr. Opin. Neurobiol. 20, 288–295. 10.1016/j.conb.2010.03.007 20493680

[B21] Manolopoulos K. Reisis D. Chouliaras V. (2016). An efficient multiple precision floating-point multiply-add fused unit. Microelectron. J. 49, 10–18. 10.1016/j.mejo.2015.10.012

[B22] Martí D. Rigotti M. Seok M. Fusi S. (2015). Energy-efficient neuromorphic classifiers. Neural Comput. 28, 2011–2044. 10.1162/NECO_a_00882 27557100

[B23] Merolla P. A. Arthur J. V. Alvarez-Icaza R. Cassidy A. S. Sawada J. Akopyan F.. (2014). A million spiking-neuron integrated circuit with a scalable communication network and interface. Science 345, 668–673. 10.1126/science.1254642 25104385

[B24] Neftci E. O. Mostafa H. Zenke F. (2019). Surrogate Gradient Learning in Spiking Neural Networks. New York, NY: IEEE.

[B25] O'Connor P. Neil D. Liu S.-C. Delbruck T. Pfeiffer M. (2013). Real-time classification and sensor fusion with a spiking deep belief network. Front. Neurosci. 7:178. 10.3389/fnins.2013.00178 24115919 PMC3792559

[B26] Orchard G. Jayawant A. Cohen G. K. Thakor N. (2015). Converting static image datasets to spiking neuromorphic datasets using saccades. Front. Neurosci. 9:437. 10.3389/fnins.2015.00437 26635513 PMC4644806

[B27] Osswald M. Ieng S.-H. Benosman R. Indiveri G. (2017). A spiking neural network model of 3d perception for event-based neuromorphic stereo vision systems. Sci. Rep. 7:40703. 10.1038/srep40703 28079187 PMC5227683

[B28] Pérez-Carrasco J. A. Zhao B. Serrano C. Acha B. Serrano-Gotarredona T. Chen S.. (2013). Mapping from frame-driven to frame-free event-driven vision systems by low-rate rate coding and coincidence processing-application to feedforward convnets. IEEE Trans. Pattern Anal. Mach. Intell. 35, 2706–2719. 10.1109/TPAMI.2013.71 24051730

[B29] Pfeiffer M. Pfeil T. (2018). Deep learning with spiking neurons: opportunities and challenges. Front. Neurosci. 12:774. 10.3389/fnins.2018.00774 30410432 PMC6209684

[B30] Posch C. Matolin D. Wohlgenannt R. (2010). High-DR frame-free PWM imaging with asynchronous AER intensity encoding and focal-plane temporal redundancy suppression, in Proceedings of 2010 IEEE International Symposium on Circuits and Systems, 2430–2433. 10.1109/ISCAS.2010.5537150

[B31] Qiao N. Mostafa H. Corradi F. Osswald M. Stefanini F. Sumislawska D.. (2015). A reconfigurable on-line learning spiking neuromorphic processor comprising 256 neurons and 128k synapses. Front. Neurosci. 9:141. 10.3389/fnins.2015.00141 25972778 PMC4413675

[B32] Rebecq H. Ranftl R. Koltun V. Scaramuzza D. (2019). Events-to-video: bringing modern computer vision to event cameras. CoRR abs/1904.08298. 10.1109/CVPR.2019.00398

[B33] Rieke F. Warland D. Ruyter R. D. Steveninck V. Bialek W. (1999). Spikes: Exploring the Neural Code. Cambridge, MA: MIT Press.

[B34] Rueckauer B. Lungu I.-A. Hu Y. Pfeiffer M. Liu S.-C. (2017). Conversion of continuous-valued deep networks to efficient event-driven networks for image classification. Front. Neurosci. 11:682. 10.3389/fnins.2017.00682 29375284 PMC5770641

[B35] Schemmel J. Brüderle D. Grübl A. Hock M. Meier K. Millner S. (2010). A wafer-scale neuromorphic hardware system for large-scale neural modeling, in Proceedings of 2010 IEEE International Symposium on Circuits and Systems (Paris), 1947–1950. 10.1109/ISCAS.2010.5536970

[B36] Shrestha S. B. Orchard G. (2018). Slayer: spike layer error reassignment in time, in Advances in Neural Information Processing Systems 31, eds Bengio S. Wallach H. Larochelle H. Grauman K. Cesa-Bianchi N. Garnett R. (Montreal, QC: Curran Associates, Inc.), 1412–1421.

[B37] Sironi A. Brambilla M. Bourdis N. Lagorce X. Benosman R. (2018). HATS: Histograms of averaged time surfaces for robust event-based object classification, in The IEEE Conference on Computer Vision and Pattern Recognition (CVPR) (Salt Lake City, UT). 10.1109/CVPR.2018.00186

[B38] Thakur C. S. Molin J. L. Cauwenberghs G. Indiveri G. Kumar K. Qiao N.. (2018). Large-scale neuromorphic spiking array processors: a quest to mimic the brain. Front. Neurosci. 12:891. 10.3389/fnins.2018.00891 30559644 PMC6287454

[B39] Tompson J. Goroshin R. Jain A. LeCun Y. Bregler C. (2015). Efficient object localization using convolutional networks, in The IEEE Conference on Computer Vision and Pattern Recognition (CVPR) (Boston, MA). 10.1109/CVPR.2015.7298664

[B40] van den Oord A. Dieleman S. Zen H. Simonyan K. Vinyals O. Graves A. (2016a). Wavenet: A generative model for raw audio. CoRR abs/1609.03499.

[B41] van den Oord A. Kalchbrenner N. Kavukcuoglu K. (2016b). Pixel recurrent neural networks. CoRR abs/1601.06759.

[B42] Wang Q. Zhang Y. Yuan J. Lu Y. (2019). Space-time event clouds for gesture recognition: From RGB cameras to event cameras, in 2019 IEEE Winter Conference on Applications of Computer Vision (WACV) (Honolulu, HI), 1826–1835. 10.1109/WACV.2019.00199

[B43] Werbos P. J. (1990). Backpropagation through time: what it does and how to do it. Proc. IEEE 78, 1550–1560. 10.1109/5.58337

[B44] Wu Y. Deng L. Li G. Zhu J. Shi L. (2018). Spatio-temporal backpropagation for training high-performance spiking neural networks. Front. Neurosci. 12:331. 10.3389/fnins.2018.00331 29875621 PMC5974215

[B45] Wu Y. Deng L. Li G. Zhu J. Xie Y. Shi L. (2019). Direct training of spiking neural networks: faster, larger, better, in Proceedings of the AAAI Conference on Artificial Intelligence (Honolulu, HI). 10.1609/aaai.v33i01.33011311

[B46] Zhang Z. Liang X. Dong X. Xie Y. Cao G. (2018). A sparse-view ct reconstruction method based on combination of densenet and deconvolution. IEEE Trans. Med. Imaging 37, 1407–1417. 10.1109/TMI.2018.2823338 29870369

[B47] Zhu Y. Newsam S. (2017). Densenet for dense flow, in 2017 IEEE International Conference on Image Processing (ICIP) (Beijing), 790–794. 10.1109/ICIP.2017.8296389

