# Supplementary material for: Efficient Processing of Spatio-Temporal Data Streams With Spiking Neural Networks
Source: Front Neurosci. 2020 May 5;14:439. doi: 10.3389/fnins.2020.00439 (PMC7214871; doi:10.3389/fnins.2020.00439)
Supplement: Supplementary file 1 [file Image_1.pdf]

frontiers

Supplementary Material

A DATASET AND TRAINING DETAILS

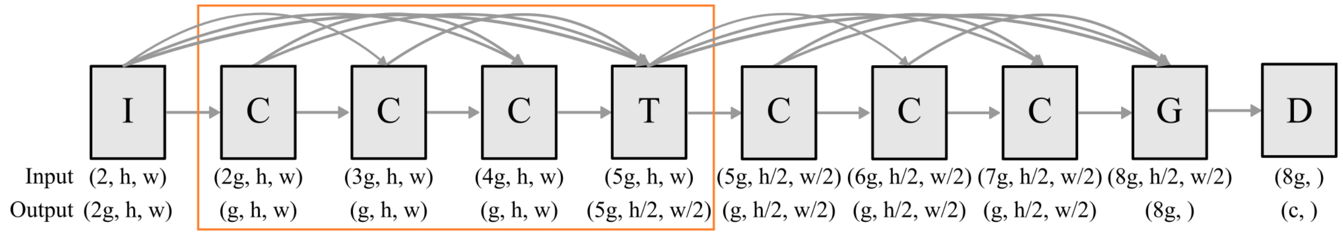

Figure S1: Connectivity of a DenseNet (Huang et al., 2017) with $N_\mathrm{l} = 3$ layers per block (orange) and $N_\mathrm{b} = 2$ blocks. The letters correspond to I: initial convolution, C: convolutional layer, T: transition layer (convolution + pooling), G: global pooling, D: fully-connected layer. Input and output shapes depend on a single hyperparameter, the growth factor g. The shapes are given as $(\mathrm{channel},\ y,\ x)$, omitting the batch dimension. The last output channel is the number of classes c. We use average pooling in all our experiments.

We use streaming rollouts of the DenseNet network model for all our experiments (see Fig. S1). At the beginning of training for N_e epochs, we set the learning rate to $l_\mathrm{r} = 0.001$. We multiply the learning rate by 0.1 after $0.6N_\mathrm{e}$ and again after $0.8N_\mathrm{e}$ epochs. After each epoch, we measure the average accuracy across all outputs $\mathbf{y}$ on our validation set and, if the value increased, we save this model as the new best model. For our results shown in the main text, we evaluate this best model on the test set. Whenever we report averages over multiple runs, each trial uses a different random number generator seed to initialize all weights. 20% of the training data are randomly selected (but fixed over the course of multiple trials) and used as a validation set to tune hyperparameters.

The following hyperparameters are used for all datasets if not denoted otherwise in the specific sections.

name	symbol	value
number of blocks	N_b	3
layers per block	N_l	5
growth rate of DenseNet	g	9
kernel size of convolution	k_x, k_y	3×3
padding		`SAME`
pooling type		Average pooling
bias		0
initialization		`he_normal`
activation function		ReLU
dropout: spatial dropout, rate		0.2
number of training epochs	N_e	300
activation decay: L2-norm, weight		$1 \cdot 10^{-4}$
weight decay: L2-norm, weight		$1 \cdot 10^{-5}$
batch size		32

A.1 Moving Rectangles dataset

This dataset contains 10000 training and 2000 test samples. Each sample consists of two images of size 10×60. In the first and second image, a rectangle of size 10×5 is placed at a random position inside the image boundaries. The relative position of the rectangles between both images defines the direction of movement (left or right). The rectangle can be placed at any position except for directly at the border of the image. The jump is then calculated by first drawing the direction (with probability 0.5 for both directions) and then determining the maximum jump distance j_{max} such that the whole rectangle is still in the image (in pixels). The actual jump value is then drawn uniformly between $[1, j_{\mathrm{max}}]$. The dataset is sparse, i.e. approximately 97.6% of the pixels are zero. No data augmentation is used.

Specific hyperparameters of the used DenseNet:

name	symbol	value
growth rate of DenseNet	g	20
layers per block	N_{l}	1
number of training epochs	N_e	100

A.2 `N-CARS`

Samples have different resolutions. First, we convert the events to frames (for details, see Section 2.3). Then, we resize the input frames to 40×30 pixels. To normalize the network input, we calculate the mean μ and standard deviation σ for each sample I (i.e., averaged over all dimensions) and scale each sample $I \to (I - \mu)/(6\sigma) + 0.5$, followed by clipping each value of I to $[0, 1]$. During training we augment single sequences of input frames by horizontal flips, and random shifts by 4 and 3 pixels in horizontal and vertical direction, respectively. Workflow of tuning the hyperparameters:

- Sweeping learning rate l_{r} over $\{0.00022, 0.00046, \mathbf{0.001}, 0.0022, 0.0046\}$: $l_{\mathrm{r}} = 0.001$ results in highest accuracy on the validation set.
- Sweeping number N of input frames over $\{1, 2, 4, \mathbf{8}, 16\}$: $N = 8$ frames are the smallest number of input frames with highest accuracy on the validation set.
- Sweeping growth rate g over $[6, \mathbf{9}, 12, 15, 18]$, i.e. the number of output channels of the convolutional layers within the DenseNet blocks: $g = 9$ channels are the smallest number of channels with highest accuracy on the validation set.
- For all above sweeps the best values from the other sweeps are used.

The best number of simulation steps per rollout frame is chosen after sweeping over $\{5, 10, 15, 20, 25, 30, 35\}$ steps using 1280 randomly chosen samples from the training set (see **Figure 1C**).

A.3 `N-MNIST`

The `N-MNIST` dataset has a spatial resolution of 34×34 pixels. No data augmentation is used.

B SYMBOL REFERENCE

symbol	name	Description
N	number of frames	Number of frames that each data sample is divided into
T_{F}	frame time	Time over which spikes are accumulated to generate a frame
N_{b}	number of blocks	Number of blocks of a network. See Fig. S1
N_{l}	layers per block	Number of layers per block. See Fig. S1
g	growth factor	Scales number of output channels in DenseNet. See Fig. S1
d_{ANN}	synaptic delay of ANN	Number of rollout frames information is delayed at each synapse
D	depth	Length of path in ANN/SNN that passes the most edges
l_{s}	shortest path	Path from one input to one output that passes the least edges
K	number of rollout frames	Number of steps from first input to last output
$\mathbf{y}_i$	network prediction	Non-normalized output of the network at rollout frame i
$T_{\max}$	sequence length	Time difference between last and first spike in a sequence
$\mathbf{F}_{\mathrm{in}}$	frames	All frames of a sample
F	frame	A frame of a sample
$\hat{\mathbf{y}}$	class label	One-hot label of a sample
l_{r}	learning rate	Learning rate during training
N_{e}	number of epochs	Number of epochs to train
a_k	loss weight	Weight assigned to each output k to tune early-to-late accuracy
ρ	accuracy ratio	Ratio of area of ANN and SNN accuracy
V_i	membrane voltage	Membrane voltage of neuron i
V_{th}	membrane threshold	Threshold at which the neurons emits a spike
d	synaptic delay of SNN	Number of simulation steps information is delayed at each synapse
r_i	neuron firing rate	Firing rate of neuron i
$r_{\max}$	maximum firing rate	Maximum possible firing rate
$\mathbf{S}$	spike train	All spikes in a defined time interval
$\mathbf{S}_{\mathrm{in}}$	spike input sequence	All spikes of a sample
n_{sf}	simulaton steps per frame	Number of simulaton steps per frame
T_{tot}	number of simulaton steps	Total number of simulaton steps
f	simulator throughput	Throughput of the simulator/emulator

REFERENCES

Huang, G., Liu, Z., and Weinberger, K. Q. (2017). Densely connected convolutional networks. In *The IEEE Conference on Computer Vision and Pattern Recognition (CVPR)* 1
